# Efficacy comparison of four different Chinese herbal mediciness in intervening acute respiratory distress syndrome: a bayesian network meta-analysis

**DOI:** 10.3389/fphar.2025.1671930

**Published:** 2025-11-21

**Authors:** Xinhui Wu, Sisi Feng, Xuemei Dai, Zhihao Liu, Yi Luo, Fei Wang

**Affiliations:** Chengdu University of Traditional Chinese Medicine, Chengdu, Sichuan, China

**Keywords:** acute respiratory distress syndrome, Chinese herbal medicines, Xuanbai chengqi decoction, fusu agent, network meta-analysis

## Abstract

**Background:**

Acute Respiratory Distress Syndrome (ARDS) is a critical condition with high ICU mortality. Despite treatments such as mechanical ventilation, outcomes remain poor, with in-hospital mortality reaching 40% and various complications negatively affecting patient prognosis. Chinese Herbal Medicines (CHM) offers a promising, safe, and reliable alternative therapy. When combined with Western symptomatic treatments, CHM can produce synergistic effects, enhancing the overall intervention and improving patient outcomes.

**Objective:**

This study aims to evaluate the efficacy of four CHMs - Xuanbai Chengqi Decoction (XBCQD), Dachengqi Decoction (DCQD), Liangge Powder (LGP), and Fusu Agent (FSA) - in managing ARDS. Using a network meta-analysis (NMA), the study ranks these formulas based on key clinical indicators, including the mechanical ventilation duration (MVD), ICU length of stay (ICU-LOS), and the PaO_2_/FiO_2_ ratio (P/F ratio).

**Results:**

A comprehensive literature review identified 18 clinical studies involving 1,134 participants to assess the relative efficacy of the four CHMs. The findings indicated that XBCQD was the most effective in reducing MVD (SUCRA = 87.8%), followed by FSA (64.3%). FSA also demonstrated superior efficacy in shortening ICU-LOS (SUCRA = 73.9%) and improving the P/F ratio (92.2%), with XBCQD ranking second in both outcomes (67.6% and 72.6%, respectively). A significant difference was found between FSA and LGP in improving the P/F ratio (MD = 41.08; 95% CrI: 5.95, 76.22). In contrast, DCQD and LGP ranked lower across most outcomes. Notably, the difference between LGP and placebo in improving oxygenation was not statistically significant (MD = 22.06; 95% CrI: −2.22, 46.35).

**Conclusion:**

In combination with Western symptomatic treatments, XBCQD is the most effective intervention for reducing MVD in ARDS patients. FSA demonstrates superior efficacy in improving the P/F ratio and shortening ICU-LOS. However, these findings require further clinical validation to establish their applicability.

## 1 Introduction

Acute Respiratory Distress Syndrome (ARDS) is a common, life-threatening condition characterized by a high mortality rate (Ware and Matthay, 2000). A variety of factors - including trauma, drug exposure, bacterial or viral pneumonia, and sepsis - can trigger acute inflammatory lung injury, ultimately culminating in ARDS (Frohlich et al., 2012). Despite advances in supportive care, there are still no specific pharmacological treatments proven effective for ARDS. Clinical management mainly depends on mechanical ventilation and other supportive measures (Beitler et al., 2022). However, a study published in *the Journal of the American Medical Association (JAMA)* reported that even with optimized ventilation strategies and adjunctive therapies such as continuous neuromuscular blockade, high-dose corticosteroids, and lung recruitment maneuvers, outcomes remain poor, with an in-hospital mortality rate of approximately 40% (Bellani et al., 2016).

In Traditional Chinese Medicine (TCM), ARDS is categorized as a “dyspnea syndrome,” with pathophysiological features such as lung qi deficiency, stagnation, and obstruction. TCM offers distinctive therapeutic advantages in managing ARDS, particularly through regulating qi flow and restoring pulmonary function, showing potential benefits beyond conventional therapies. In clinical settings, the integration of TCM syndrome differentiation and treatment with Western ventilatory support has demonstrated synergistic effects, providing complementary advantages and improving overall treatment efficacy (Guo et al., 2024).

A comprehensive literature review indicates that the primary Chinese Herbal Mediciness (CHMs) currently employed in the treatment of ARDS include Xuanbai Chengqi Decoction (XBCQD), Dachengqi Decoction (DCQD), Liangge Powder (LGP), and Fusu Agent (FSA). These formulas have shown promising therapeutic effects in clinical practice (Ma et al., 2022).

Despite these encouraging findings, existing studies have not yet clarified which formula provides the greatest therapeutic benefit, creating uncertainty for clinicians when selecting the optimal intervention. Therefore, the present study employs a network meta-analysis (NMA) to compare the efficacy of these four CHM formulas in managing ARDS. The treatments are ranked according to key clinical outcomes, including mechanical ventilation duration (MVD), PaO_2_/FiO_2_ (P/F ratio), and ICU length of stay (ICU-LOS), with the aim of informing evidence-based decision-making in clinical practice.

## 2 Materials and methods

This study adhered to the Preferred Reporting Items for Systematic Reviews and Meta-Analyses (PRISMA) extended statement guidelines (Supplementary Table S1 for details) (Page et al., 2021). The study is registered under the International Prospective Register of Systematic Reviews (PROSPERO: https://www.crd.york.ac.uk/PROSPERO/) with the registration number CRD420251076146. Given that all analyses were built upon previously published research, ethical approval and patient consent were deemed unnecessary for this investigation.

### 2.1 Search strategy

The comprehensive search encompassed multiple databases, including Web of Science, PubMed, EMBASE, Cochrane Central Controlled Trials, China Knowledge Network (CNKI), Wanfang database, VIP database, and China Biomedical Literature Database (CBM). The search targeted RCTs assessing the effectiveness of four traditional Chinese herbal medicines (XBCQD, DCQD, LGP, and FSA) in ARDS up to 15 June 2025. Language or publication date limitations were not imposed.

Utilizing a meticulous approach, the search strategy integrated Medical Subject Headings (MeSH) and free words, employing Boolean logic operators. The key terms incorporated for the comprehensive search were “acute respiratory distress syndrome,” “ARDS,” “Xuanbai Chengqi Decoction,” “Dachengqi Decoction,” “Liangge Powder,” “Fusu Agent,” and “randomized controlled trial.” Specific details of the search strategy, exemplified by the PUBMED search, are provided in Supplementary Table S2. Two independent authors, Xinhui Wu and Xuemei Dai, conducted the screening using Endnote 20 literature management software (Thompson ISI Research Soft, Philadelphia, Pennsylvania, USA). Any discrepancies during this process were resolved through consensus or consultation with a third author, Wang Fei, ensuring the reliability and transparency of the literature selection process.

### 2.2 Selection and exclusion criteria

The study selection adhered to the PICOS framework:

Population: Patients diagnosed with acute respiratory distress syndrome (ARDS) according to well-defined diagnostic criteria (Chinese Society of Critical Care Medicine, 2007), without restrictions on gender or age.

Intervention: Four Chinese herbal medicine (CHM) interventions were included - Xuanbai Chengqi Decoction (XBCQD), Dachengqi Decoction (DCQD), Liangge Powder (LGP), and Fusu Agent (FSA).

Comparison: Comparisons encompassed placebo or intercomparisons between CHM interventions. All groups received standard biomedical treatments, including management of underlying diseases, mechanical ventilation, fluid resuscitation, antimicrobial therapy, vasoactive agents, correction of acidosis, maintenance of fluid, electrolyte, and acid - base balance, nutritional support, and comprehensive organ function support.

Outcomes: The primary outcome was mechanical ventilation duration (MVD), while secondary outcomes included ICU length of stay (ICU-LOS) and PaO_2_/FiO_2_ (P/F ratio).

Timing: Only studies reporting treatment duration sufficient to evaluate the above outcomes were included.

Setting: Studies conducted in hospital or ICU settings were eligible.

Study design: Only randomized controlled trials (RCTs) were included. Non-RCTs, reviews, systematic reviews, case-control studies, and study protocols were excluded.

Utilizing these criteria, two authors (X.W. and X.D.) independently screened titles and abstracts, removed duplicates and ineligible studies, and reviewed full texts for inclusion. Discrepancies were resolved through discussion or consultation with a third reviewer to ensure a rigorous and systematic selection process.

### 2.3 Data extraction and quality assessment

Relevant information from eligible studies was systematically gathered utilizing the Cochrane Consumer and Communications Review Group’s data extraction template. This comprehensive data collection encompassed essential publication details, participant characteristics (total sample size, age, and disease duration), intervention specifics, treatment duration, and the quality assessment of RCTs, along with other pertinent data.

Two independent researchers, ZHL and YL, rigorously assessed the quality of each eligible study utilizing the Cochrane Risk of Bias Tool (Savović et al., 2014). This tool, applied across seven domains, facilitated a thorough evaluation of each project’s risk of bias, categorized as unknown, low, or high. The quality assessment process was executed using Review Manager (version 5.4), ensuring a standardized and reliable evaluation of study quality. Any discrepancies during the assessment were resolved through consensus, maintaining the integrity and robustness of the quality evaluation process.

### 2.4 Statistical analyses

Utilizing minimally informative prior distributions in the Bayesian random effects model (Mengersen and Stojanovski, 2006), we initially conducted a conventional pair-wise meta-analysis, amalgamating crucial data from all included studies. Mean differences (MDs) with corresponding 95% credible intervals (CrIs) were used to estimate pooled effect sizes. Statistical heterogeneity was quantified using the I^2^ statistic, and potential publication bias was explored through comparison-adjusted funnel plots and Egger’s regression test (Melsen et al., 2014; Seagroatt and Stratton, 1998). A network plot was generated to visualize treatment relationships, with different interventions represented as nodes and individual trials as connecting lines. The evaluation of network transitivity is pivotal in NMA and significantly influences subsequent analyses (Salanti, 2012). To ensure the comparability of different treatments and the validity of drawing indirect conclusions, we scrutinized the transitivity assumption. This involved a meticulous comparison of clinical and methodological characteristics, including participant attributes and experimental design, across all included studies (Caldwell et al., 2005; Jansen and Naci, 2013). For precise estimation of the statistical model, we established four parallel Markov chains in the random selection state (Mavridis and Salanti, 2013). Each chain underwent 20,000 iterations, with an initial burn-in period discarding the first 5,000 iterations. This practice minimized bias from initial values when the chain reached its target distribution (Valkenhoef and Kuiper, 2015). Convergence evaluation employed the Brooks-Gelman-Rubin diagnostic, involving a visual inspection of the historical trajectory of trace combined with density plots (Brooks and Gelman, 1998). (Supplementary Figure S1 for details). Treatment hierarchies were ranked using the Surface Under the Cumulative Ranking Curve (SUCRA), with higher SUCRA values indicating greater treatment efficacy (Page et al., 2016).

All analyses were performed using R (version 4.5.1; gemtc version 1.1–0, rjags version 4-17) and STATA (version 18.0, StataCorp, College Station, TX, USA). The methodological framework builds upon our previously published work (Feng et al., 2023), refined to align with the specific objectives of this study.

## 3 Results

### 3.1 Search process and baseline characteristics

**FIGURE 1:**
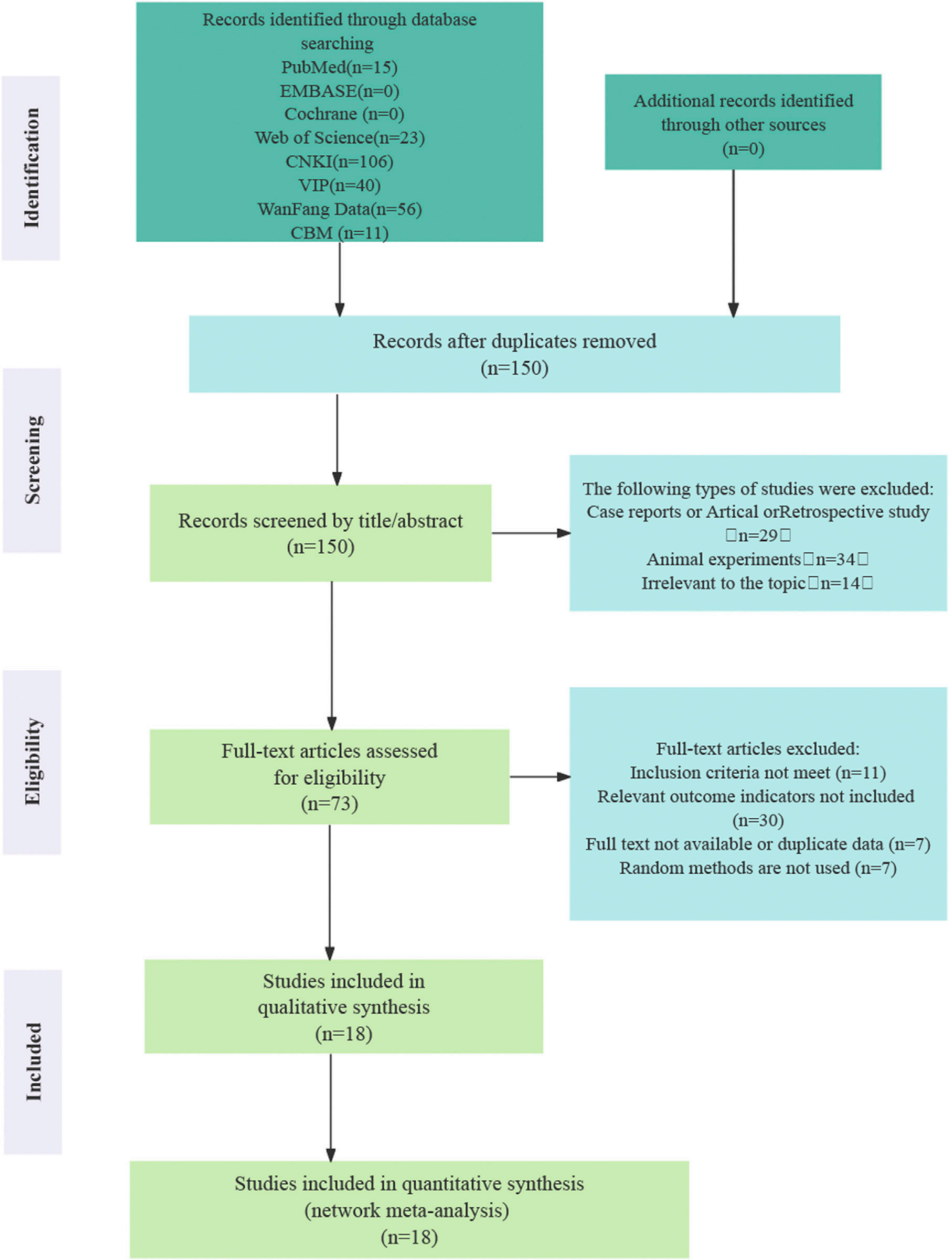
Literature screening process.

The initial search yielded 251 records, of which 101 were identified as duplicates and removed. After screening titles and abstracts, 77 articles were excluded as they were review articles, retrospective studies, or animal experiments. Full-text assessments were conducted on the remaining 73 articles, resulting in the exclusion of 11 studies that did not meet the inclusion criteria. An additional 30 studies were excluded due to the absence of relevant outcome indicators, seven lacked randomization, and seven were excluded due to unavailability of the full text or duplicated data. Ultimately, 18 randomized controlled trials (RCTs) that fulfilled all inclusion criteria were included in the final analysis (Xia and Yan, 2025; LU and Zheng, 2023; Zeng et al., 2023; Zhang and Mao, 2016; Yi et al., 2019; Liu, 2019; Chen, 2018; Lu et al., 2017; Wang et al., 2015; Li et al., 2024a; Lu et al., 2018; Liu, 2014; Su, 2011; Dai et al., 2018; Ding, 2020; Zhang et al., 2020a; Lei et al., 2018; Guo, 2014). Figure 1 illustrates the process of literature screening.

Table 1 presents a comprehensive overview of essential features, including participant baseline characteristics and interventions. A total of 1,134 participants were randomly assigned to trial or control groups. Among them, 570 participants were in the trial group, undergoing four different traditional CHM interventions (XBCQD, n = 138; DCQD, n = 174; LGP, n = 123; FSA, n = 135). The remaining 564 individuals were randomized into the control group (i.e., the placebo group).

**TABLE 1:** Characteristic of included studies.

Study ID	N (T/C)	Age	Gender (M/F)	Route of administration	Interventions (All participants received standard treatment for ARDS)		Course	Outcome
					T	C		
Xia and Yan (2025)	60/60	T: 50.28 ± 2.49 C: 50.28 ± 2.49	T: 38/22 C: 35/25	NGT	XBCQD	Placebo	7d	①②③
Lu and Zheng (2023)	30/30	T: 65.33 ± 6.44 C: 63.30 ± 8.59	T: 18/12 C: 16/14	PO/NGT	XBCQD	Placebo	7d	①②③
Zeng et al. (2023)	33/33	T: 51.34 ± 7.24 C: 53.29 ± 8.35	T: 18/15 C: 20/13	NGT	XBCQD	Placebo	7d	②③
Zhang and Mao (2016)	15/15	T: 53.0 ± 9.6 C: 52.3 ± 8.8	T: 11/4 C: 10/5	PR	XBCQD	Placebo	7d	①③
Yi et al. (2019)	45/45	T: 46.32 ± 9.85 C: 45.62 ± 10.37	T: 24/21 C: 23/22	PR	DCQD	Placebo	7d	①②③
Liu (2019)	20/20	T: 63.68 ± 11.56 C: 62.00 ± 12.81	T: 13/7 C: 9/11	NGT	DCQD	Placebo	5d	①③
Chen (2018)	38/38	T: 61.02 ± 3.56 C: 61.08 ± 3.59	-	PR	DCQD	Placebo	5d	①③
Lu et al. (2017)	41/41	T: 47.12 ± 6.75 C: 47.69 ± 6.82	T: 22/19 C: 24/17	NGT	DCQD	Placebo	7d	①②③
Wang et al. (2015)	30/30	T: 46.12 ± 6.75 C: 45.80 ± 11.6	T: 16/14 C: 17/13	PR	DCQD	Placebo	7d	②
Li et al. (2024a)	50/50	T: 61.12 ± 7.04 C: 59.16 ± 7.02	T: 28/22 C: 26/24	PO/NGT	LGP	Placebo	14d	①②③
Lu et al. (2018)	15/15	T: 55.13 ± 15.68 C: 58.80 ± 18.66	T: 8/7 C: 7/ 8	PR	LGP	Placebo	7d	①②③
Liu (2014)	15/15	T: 59.90 ± 14.80 C: 60.60 ± 10.7	T: 9/6 C: 8/7	NGT/PR	LGP	Placebo	7d	②
Su (2011)	15/14	T: 49.13 ± 17.25 C: 56.14 ± 14.98	T: 8/7 C: 9/4	PO/NGT	LGP	Placebo	7d	②
Dai et al. (2018)	28/24	T: 61.08 ± 11.64 C: 62.46 ± 12.04	T: 14/14 C: 12/12	PO/NGT	LGP	Placebo	7d	②
Ding (2020)	30/30	T: 59.60 ± 11.84 C: 55.45 ± 13.55	26/34	PO/NGT	FSA	Placebo	14d	②③
Zhang et al. (2020a)	25/25	T: 58.30 ± 11.50 C: 56.90 ± 9.7	T: 15/10 C: 13/12	NGT	FSA	Placebo	14d	①②③
Lei et al. (2018)	50/50	T: 43.35 ± 3.11 C: 43.26 ± 3.07	T: 28/22 C: 30/20	NGT	FSA	Placebo	5d	②
Guo (2014)	30/29	T: 65.43 ± 8.24 C: 64.38 ± 7.26	T: 18/12 C: 19/10	PO/NGT	FSA	Placebo	14d	①②③

① Duration of mechanical ventilation; ② PaO/FiO ratio; ③ ICU length of stay.

T, Treatment group; C, Control group; PR, Rectal enema; NGT, Nasogastric administration; PO, Oral administration; XBCQD, Xuanbai Chengqi Decoction; DCQD, Dachengqi Decoction; LGP, Liangge Powder; FSA, Fusu Agent.

#### 3.1.1 Quality of included studies

The risk of bias plot and summary are provided in Supplementary Figure S2. All 18 (Xia and Yan, 2025; LU and Zheng, 2023; Zeng et al., 2023; Zhang and Mao, 2016; Yi et al., 2019; Liu, 2019; Chen, 2018; Lu et al., 2017; Wang et al., 2015; Li et al., 2024a; Lu et al., 2018; Liu, 2014; Su, 2011; Dai et al., 2018; Ding, 2020; Zhang et al., 2020a; Lei et al., 2018; Guo, 2014) studies included comprehensive data on the pre-specified outcome measures. Of these, 16 studies (Xia and Yan, 2025; LU and Zheng, 2023; Zeng et al., 2023; Zhang and Mao, 2016; Yi et al., 2019; Liu, 2019; Chen, 2018; Wang et al., 2015; Zhang et al., 2020a; Guo, 2014) clearly outlined their randomization methods, while two studies (Lu et al., 2017; Lei et al., 2018) only mentioned 'random.' Furthermore, two studies (Ding, 2020; Guo, 2014) implemented allocation concealment and blinding procedures, and eight studies (Zeng et al., 2023; Yi et al., 2019; Chen, 2018; Lu et al., 2017; Li et al., 2024a; Ding, 2020; Guo, 2014) offered detailed information on patient dropout, adverse events, or follow-up data.

### 3.2 Conventional meta-analysis results

The preliminary conventional meta-analysis of the MVD, ICU LOS, and P/F ratio outcomes revealed substantial heterogeneity (MVD: I^2^ = 86.5%, *P* < 0.001; ICU LOS: I^2^ = 93.8%, *P* < 0.001; P/F ratio: I^2^ = 88.7%; *P* < 0.001); (Supplementary Figure S3a,b,c). To investigate the sources of this heterogeneity and evaluate the robustness of the findings, we performed additional analyses, including meta-regression, Egger’s test for publication bias, sensitivity analysis, and adjusted funnel plots. The detailed results of these analyses are provided in the “Heterogeneity Assessment and Analysis” section.

### 3.3 Network analysis results

#### 3.3.1 Primary outcome: mechanical ventilation duration (MVD)

**FIGURE 2:**
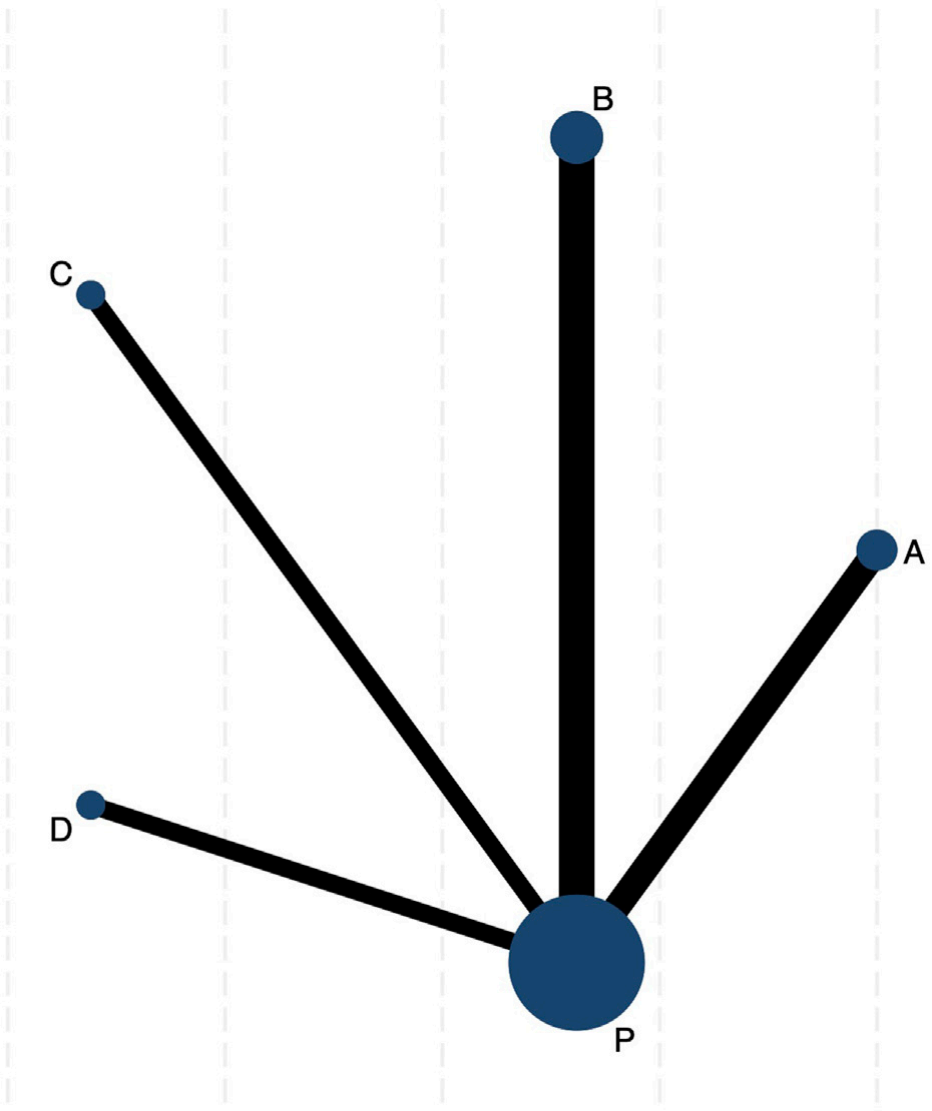
The network evidence graph of mechanical ventilation duration (MVD). P. Placebo; **(A)** Xuanbai Chengqi Decoction (XBCQD); **(B)** Dachengqi Decoction (DCQD); **(C)** Liangge Powder (LGP) **(D)**. Fusu Agent (FSA).

**FIGURE 3:**
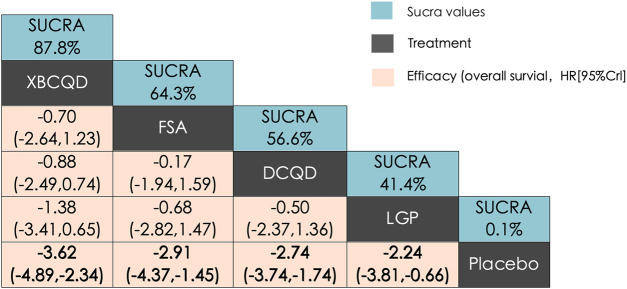
League matrix table of Mechanical Ventilation Duration (MVD) Treatments were ranked in order of their likelihood of being the best treatment. The numbers in the green boxes are SUCRA values, representing the rank of the treatments. Meaningful pairwise comparisons are highlighted in bold. P. Placebo; **(A)** Xuanbai Chengqi Decoction (XBCQD); **(B)** Dachengqi Decoction (DCQD); **(C)** Liangge Powder (LGP) **(D)**. Fusu Agent (FSA).

Figure 2 illustrates a network diagram consisting of four intervention groups and one placebo control group. Each node represents a specific intervention, and its size correlates with the number of patients studied. Specifically, DCQD (n = 144) was involved in four arms, XBCQD (n = 105) in three arms, and both LGP (n = 65) and FSA (n = 55) and in two arms each. Figure 3 illustrates the effectiveness of various CHM interventions in reducing the mechanical ventilation duration (MVD). All four interventions significantly reduced DMV compared with the control group, as supported by SUCRA rankings (Figures 3, 6a). Among them, XBCQD demonstrated the greatest efficacy (SUCRA = 87.8%), followed by FSA (64.3%), DCQD (56.6%), and LGP (41.4%).

### 3.4 Secondary outcome

#### 3.4.1 ICU length of stay (ICU LOS)

**FIGURE 4:**
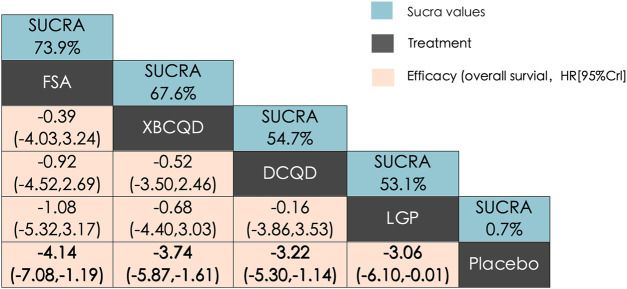
League matrix table of ICU length of stay (ICU LOS). Treatments were ranked in order of their likelihood of being the best treatment. The numbers in the green boxes are SUCRA values, representing the rank of the treatments. Meaningful pairwise comparisons are highlighted in bold. P. Placebo; **(A)** Xuanbai Chengqi Decoction (XBCQD); **(B)** Dachengqi Decoction (DCQD); **(C)** Liangge Powder (LGP) **(D)** Fusu Agent (FSA).

The network structure for ICU length of stay (LOS) was comparable to that of DMV, involving the same CHM interventions and placebo (Supplementary Figure S4a). In this network, XBCQD (n = 139) and DCQD (n = 144) were each included in four study arms, FSA (n = 85) in three arms, and LGP (n = 65) in two arms. Figure 4 summarizes the effects of these interventions on ICU length of stay (LOS). A similar trend was observed: all four CHM interventions demonstrated superior outcomes compared to placebo (SUCRA = 0.7%), with FSA ranking first (SUCRA = 73.9%), followed by XBCQD (67.6%), DCQD (54.7%), and LGP (53.1%), as shown in the league table and SUCRA rankings (Figures 4, 6b). Interestingly, the ranking order differed slightly from that of DMV, with FSA outperforming XBCQD in shortening ICU LOS.

#### 3.4.2 PaO_2_/FiO_2_ ratio (P/F ratio)

**FIGURE 5:**
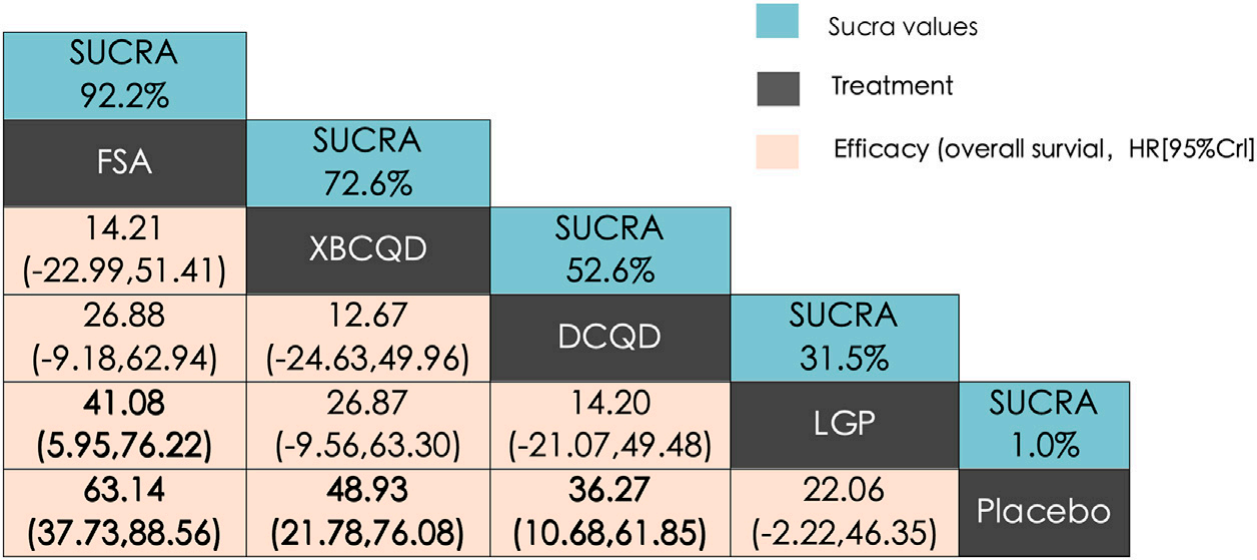
League matrix table of PaO_2_/FiO_2_ ratio (P/F ratio). Treatments were ranked in order of their likelihood of being the best treatment. The numbers in the green boxes are SUCRA values, representing the rank of the treatments. Meaningful pairwise comparisons are highlighted in bold. P. Placebo; **(A)** Xuanbai Chengqi Decoction (XBCQD); **(B)** Dachengqi Decoction (DCQD); **(C)** Liangge Powder (LGP) **(D)**. Fusu Agent (FSA).

**FIGURE 6:**
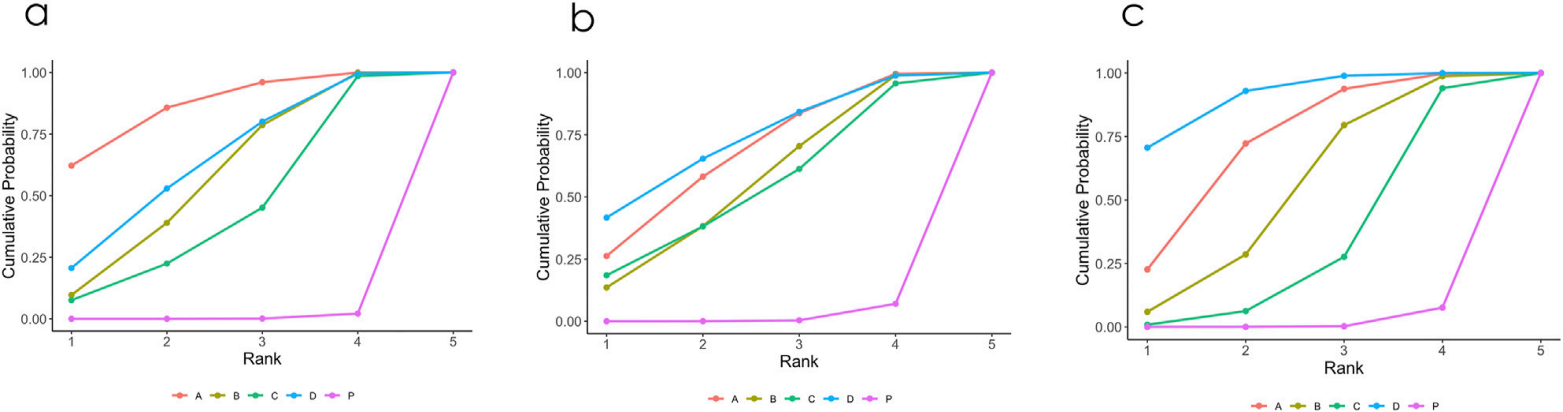
**(a–c)** Probability ranking results of different interventions. **(a)** Mechanical Ventilation Duration (MVD); **(b)** ICU length of stay (ICU LOS); **(c)** PaO_2_/FiO_2_ ratio (P/F ratio). P. Placebo; A Xuanbai Chengqi Decoction (XBCQD); B Dachengqi Decoction (DCQD); C Liangge Powder (LGP) D Fusu Agent (FSA).

A similar network of evidence was constructed for the outcome of PaO_2_/FiO_2_ ratio, comprising four CHM interventions and a placebo group (Supplementary Figure S4b). LGP (n = 123) was involved in five study arms, FSA (n = 135) in four arms, and both XBCQD (n = 124) and DCQD (n = 116) in three arms each. Figure 5 illustrates the comparative effects of the included CHM interventions on the P/F ratio. FSA, XBCQD, and DCQD demonstrated significant improvements compared to placebo, with SUCRA values of 92.2%, 72.6%, and 52.6%, respectively (Figures 5, 6c). Notably, LGP (SUCRA = 31.5%) did not show a statistically significant advantage over placebo in improving oxygenation (MD = 22.06; 95% CrI: −2.22, 46.35). Furthermore, FSA was significantly more effective than LGP (MD = 41.08; 95% CrI: 5.95, 76.22), highlighting its superior efficacy in enhancing the P/F ratio.

### 3.5 Inconsistency analysis

The node-splitting method was employed to evaluate inconsistency between direct and indirect comparisons across all outcomes. No significant inconsistency was observed in the networks for the mechanical ventilation duration (MVD), ICU length of stay (ICU-LOS), or the PaO_2_/FiO_2_ ratio, indicating a high level of agreement between sources of evidence. The corresponding results are presented in Supplementary Figure S5a,b,c (*P* > 0.05).

### 3.6 Sensitivity analysis and meta regression

To explore the sources of heterogeneity for the three outcome measures, several analyses were performed. Initially, the adjusted funnel plots show a relatively symmetrical distribution, with some studies scattered at the bottom, which may indicate a potential risk of publication bias (Supplementary Figure S6a,b,c). However, in the meta-regression, intervention duration and administration method were included as covariates. The results showed that neither of these factors significantly contributed to the heterogeneity observed across studies (*P* > 0.05) (Supplementary Tables S3a,b,c). Furthermore, Egger’s test for publication bias revealed no significant evidence of publication bias (*P* > 0.05), suggesting that publication bias is unlikely to affect the overall findings (Supplementary Tables S4a,b,c). Additionally, sensitivity analysis further confirmed the stability of the results, with no single study exerting a disproportionate influence on the overall outcomes (Supplementary Figure S7a,b,c).

## 4 Discussion

This study employed a network meta-analysis (NMA) to systematically assess the relative efficacy of four commonly used Chinese herbal medicine (CHM) formulas - Xuanbai Chengqi Decoction (XBCQD), Dachengqi Decoction (DCQD), Liangge Powder (LGP), and Fusu Agent (FSA) - when used in combination with conventional Western symptomatic treatment for acute respiratory distress syndrome (ARDS). The objective was to evaluate the comparative efficacy and clinical applicability of different Traditional Chinese Medicine (TCM) intervention strategies.

The results demonstrated that XBCQD was the most effective in reducing the duration of invasive mechanical ventilation (SUCRA = 87.8%), followed by FSA (64.3%). FSA showed superior performance in shortening ICU length of stay (ICU LOS) (SUCRA = 73.9%) and improving the PaO_2_/FiO_2_ ratio (P/F ratio) (SUCRA = 92.2%), with XBCQD ranking second in both outcomes (67.6%, 72.6%, respectively). A statistically significant difference was observed between FSA and LGP in improving the P/F ratio (MD = 41.08; 95% CrI: 5.95, 76.22). In contrast, DCQD and LGP ranked lower across most endpoints. Notably, the difference between LGP and placebo in oxygenation improvement was not statistically significant (MD = 22.06; 95% CrI: −2.22, 46.35). These findings suggest that different CHM interventions may have distinct therapeutic emphases in treating ARDS, indicating their potential for targeted clinical application. The variations in rankings across multiple outcome measures further validate the sensitivity and clinical relevance of the selected endpoints, offering a more comprehensive evaluation of each intervention’s therapeutic efficacy across different dimensions.

ARDS is a life-threatening condition and a major cause of ICU mortality. At present, there is a lack of specific and effective therapies. Although mechanical ventilation and fluid management constitute the cornerstone of standard supportive treatment, associated complications - such as ventilator-associated lung injury and multiple organ dysfunction - can severely impact patient outcomes (Slutsky, 2015; Fan et al., 2018). Against this background, TCM, with its unique advantages of multi-target and systemic regulatory effects, offers a promising new perspective for the comprehensive management of ARDS. Emerging evidence suggests that CHM exerts beneficial effects in improving oxygenation and modulating inflammatory responses (Zhang et al., 2020b; Li et al., 2022). When used in combination with standardized Western medical treatment, CHM may further enhance clinical outcomes, underscoring the therapeutic potential of integrative interventions that unite traditional Chinese and Western medicine approaches.

In parallel, selecting appropriate outcome measures is essential for accurately evaluating treatment efficacy and ensuring the methodological quality of clinical research. Ideal endpoints should be closely linked to patient prognosis and quality of life, serving as critical indicators of the scientific rigor of randomized controlled trials (RCTs) (Huang et al., 2020). In this study, the mechanical ventilation duration (MVD) was designated as the primary outcome, reflecting the recovery of pulmonary function and the process of weaning. ICU LOS and P/F ratio were selected as secondary outcomes to evaluate disease progression, resource utilization, and improvement in oxygenation, respectively. These endpoints were selected with reference to the *Expert Consensus on the Standardized Data Set for ARDS (2022 Edition)* (Expert Panel on the Standardized, 2022), ensuring high reliability and clinical relevance. Furthermore, the use of NMA overcomes the limitations of traditional pairwise meta-analyses in comparing multiple interventions simultaneously (Lumley, 2002). To our knowledge, this study represents the first comprehensive comparison of several commonly used CHM interventions for ARDS. By identifying their respective therapeutic emphases and areas of advantage, it provides valuable evidence for guiding the optimal selection of TCM strategies in integrative ARDS treatment.

Xuanbai Chengqi Decoction (XBCQD), originating from *Wenbing Tiaobian* (*Treatise on Differentiation and Treatment of Warm Diseases*), is composed of *Armeniacae Semen* (Xingren), *Gypsum Fibrosu*m (Shigao), *Rhei Radix et Rhizoma* (Dahuang), and *Trichosanthis Pericarpium* (Gualoupi). In this formula, *Gypsum Fibrosum* serves as the principal (jun) herb, clearing heat and purging fire. *Rhei Radix et Rhizoma* functions to unblock the bowels and eliminate internal heat, promoting downward discharge. *Armeniacae Semen* disseminates lung qi, redirects rebellious qi downward, relieves chest oppression, and suppresses cough. *Trichosanthis Pericarpium* clears the lungs, moistens dryness, resolves phlegm, and expands the chest. The synergistic combination of these four herbs embodies the therapeutic principle of “treating the Lung via the Fu-organ (Large Intestine)”, simultaneously addressing heat clearance, bowel purgation, lung qi diffusion, and phlegm resolution. This aligns with the treatment strategy grounded in the Lung-Large Intestine exterior–interior relationship, a core tenet in TCM. As stated in the *Lingshu* (*Spiritual Pivo*t), “The Lung is internally-externally related to the Large Intestine.” The downward flow of Lung qi facilitates intestinal peristalsis, while unobstructed bowel movement in turn supports the ascent of Lung qi (Ding, 2004; Li et al., 2024b; Liu et al., 2011).

Recent studies have identified immune dysregulation - induced acute pulmonary inflammation as the core pathological mechanism underlying ARDS (Bos and Ware, 2022). Various inflammatory cells, including neutrophils and macrophages, accumulate in the lung tissue and activate the TLR4/NF-κB signaling pathway, leading to the release of pro-inflammatory cytokines such as IL-6, TNF-α, and IL-1β. This cascade causes damage to alveolar epithelial cells and capillary endothelial cells, resulting in diffuse alveolar exudation, pulmonary edema, and severe hypoxemia (Dolinay et al., 2012). The recently proposed “gut–lung axis” theory (Budden et al., 2017) further reveals that gut microbiota dysbiosis can aggravate pulmonary inflammation by activating similar immune pathways and triggering oxidative stress. Translocation of gut-derived bacteria and endotoxins into the lungs via the circulatory system can initiate a cascade inflammatory response, accelerating the progression of ARDS (Wang et al., 2022; Tang et al., 2021; Dang and Marsland, 2019; Dickson et al., 2016; Dickson, 2018). Pharmacological studies have demonstrated that XBCQD exerts notable anti-inflammatory and immunomodulatory effects. Extracts of *Rhei Radix et Rhizoma* (e.g., emodin) inhibit the NF-κB pathway, reduce the release of inflammatory mediators, regulate pulmonary water metabolism, and help maintain surfactant function (Dong et al., 2016; Xiao et al., 2014; Shen et al., 2020). *Gypsum Fibrosum* contains calcium and magnesium ions that contribute to anti-inflammatory, heat-clearing, and immunomodulatory effects (Shi et al., 2021). *Armeniacae Semen* exhibits antioxidant properties, improves vascular endothelial function, and suppresses airway inflammation (Zhao et al., 2021), while *Trichosanthis Pericarpium* alleviates mucosal damage in the respiratory tract (Lei et al., 2021). These pharmacological actions closely correlate with the inflammatory pathways mediated by the gut-lung axis, suggesting a potential synergistic effect in the intervention of ARDS. Findings from our NMA further validate that XBCQD significantly shortens mechanical ventilation duration, thereby reinforcing both the mechanistic rationale and clinical relevance of its Lung-Intestine co-treatment strategy in managing ARDS.

This study also found that Fusu Agent (FSA) was the most effective in shortening ICU LOS and improving the P/F ratio, highlighting its potential advantage in enhancing pulmonary function and accelerating recovery. FSA is formulated based on the principle of “warming the kidney and subduing hyperactive yang, restoring yang to counteract collapse,” and is a modified version of Qianyang Dan, with the addition of *Zingiberis Rhizoma* (Ganjiang) and *Ephedrae Herba* (Mahuang) to enhance its therapeutic effects. Within the formula, *Aconiti Lateralis Radix Praeparata* (Fuzi), Ganjiang, and *Glycyrrhizae Radix* (Gancao) form the classical Sini Decoction, which serves to restore yang and prevent collapse. *Amomi Fructus* (Sharen) assists in grasping qi and directing it to the kidney, while also harmonizing the middle jiao. *Testudinis Plastrum* (Guiban) nourishes yin, anchors yang, and supports yang restoration. *Ephedrae Herba* disseminates lung qi, alleviates dyspnea, promotes urination, and reduces edema. The formula as a whole emphasizes warming and tonifying, with restoration of kidney yang as the foundation, facilitation of qi reception as the therapeutic priority, and relief of dyspnea as a supportive function, supplemented by regulation of water metabolism. Together, these elements constitute a comprehensive therapeutic system characterized by “restoring yang to prevent collapse and relieving dyspnea to counteract reversal.” As noted in the *Huangdi Neijing* (*The Yellow Emperor’s Inner Canon*), “Where pathogenic factors accumulate, there must be a deficiency of *zheng qi* (vital qi).” In TCM, ARDS is classified as an acute, critical, and severe syndrome (ji, wei, zhong). Although its clinical presentation often reflects excess patterns, the underlying pathogenesis is rooted in a sudden collapse of *zheng qi*, with deficiency as the fundamental cause. Based on this understanding, modern TCM scholars have proposed the theory of “acute deficiency syndrome” (Fang et al., 2017; Fang et al., 2021), which posits that ARDS essentially results from a sudden exhaustion of *zheng qi* and depletion of *yuan yang*, manifesting as a “deficiency complicated by excess” pattern. This theory underscores the TCM tenet that, even in acute conditions, treatment may be directed at the root (*zhi ben*), rather than exclusively at the manifestation (*zhi biao*). FSA aligns closely with this theoretical framework in both its formulation and therapeutic strategy, thereby providing a strong theoretical foundation for its clinical application in the treatment of ARDS.

Modern pharmacological research has further validated the intervention mechanism of the Resuscitation Formula at the molecular level. Experiments have shown that the Resuscitation Formula exerts protective effects in LPS-induced acute lung injury models in rats and human umbilical vein endothelial cells by inhibiting the expression of acetylheparinase. This action helps maintain mitochondrial transmembrane potential and alleviates cell damage, thereby improving overall survival status (Gao et al., 2018). Additionally, other studies have indicated that pretreatment with the Resuscitation Formula significantly reduces the levels of tumor necrosis factor-α (TNF-α) and intercellular adhesion molecule-1 (ICAM-1) in rats, while inhibiting the infiltration of inflammatory cells and the adhesion of neutrophils (Gao et al., 2019). These findings not only deepen our understanding of the potential mechanisms of the Resuscitation Formula but also provide scientific support for its clinical translation in the treatment of ARDS.

### 4.1 Limitations

This study has several limitations that should be acknowledged. First, the number of included trials was relatively small, and direct head-to-head comparisons among the evaluated CHMs were lacking, which may have limited the robustness and stability of the network estimates. Second, although no major source of heterogeneity or inconsistency was identified, and a random-effects model was employed to account for potential between-study variation, residual heterogeneity cannot be completely ruled out. Subtle differences in patient characteristics, intervention implementation, or study quality may still have influenced the pooled estimates. Third, while the 28-day mortality rate represents a key prognostic endpoint in ARDS, only a few eligible studies reported this outcome, restricting a comprehensive assessment of the impact of CHM on survival and long-term recovery. Overall, these limitations suggest that the present findings should be interpreted with caution. Further large-scale, multicenter, and methodologically rigorous randomized controlled trials are warranted to validate these results and strengthen the evidence base for integrating CHM into the evidence-based management of ARDS.

## 5 Conclusion

This study systematically evaluated the relative efficacy of four commonly used CHMs in combination with conventional Western symptomatic treatment for the management of ARDS using NMA. The results indicated that XBCQD demonstrated the greatest efficacy in reducing the MVD, underscoring its potential in facilitating ventilator weaning and promoting pulmonary function recovery. FSA showed the best performance in shortening ICU-LOS and improving the P/F ratio, suggesting its potential to accelerate recovery and enhance oxygenation.

Furthermore, this study revealed variability in the efficacy of different CHM interventions for ARDS, highlighting their multi-target and multi-pathway therapeutic characteristics, which may enable the development of tailored combination therapies based on clinical needs. The findings provide preliminary evidence supporting the efficacy of CHM interventions in ARDS management and offer a reference for optimizing herbal treatment regimens and advancing personalized therapeutic strategies.

Future studies - particularly large-scale, high-quality randomized controlled trials - are warranted to further validate these conclusions. In addition, mechanistic investigations using multi-omics technologies could facilitate a deeper understanding of CHM pharmacodynamics and promote the systematic integration and translational application of TCM in critical care settings.

## Data Availability

The original contributions presented in the study are included in the article/Supplementary Material, further inquiries can be directed to the corresponding author.

## Abbreviations

ARDS, Acute Respiratory Distress Syndrome; CHMs, Chinese Herbal Medicines; XBCQD, Xuanbai Chengqi Decoction; DCQD, Dachengqi Decoction; LGP, Liangge Powder; FSA, Fusu Agent; NMA, Network meta-analysis; MVD, Mechanical ventilation duration; P/F ratio, PaO2/FiO2 ratio; ICU-LOS, ICU length of stay; TCM, Traditional Chinese Medicine; RCTs, Randomized controlled trials.
