# Supplementary material for: Efficacy comparison of four different Chinese herbal mediciness in intervening acute respiratory distress syndrome: a bayesian network meta-analysis
Source: Front Pharmacol. 2025 Nov 21;16:1671930. doi: 10.3389/fphar.2025.1671930 (PMC12678923; doi:10.3389/fphar.2025.1671930)
Supplement: Supplementary file 1 [file Supplementaryfile1.docx]

Supplementary File 1. Summary Analysis Report

**1. Overview of the Analytical Framework**

This supplementary analysis report provides a detailed description of the statistical framework, model specifications, and results of the consistency assessments used in the Bayesian network meta-analysis (NMA) of Chinese herbal medicine (CHM) interventions for acute respiratory distress syndrome (ARDS).

The analysis followed a prospectively designed protocol structured according to the PICOTS framework, and all statistical procedures were prespecified to ensure methodological transparency and reproducibility.

**2. Model Structure and Prior Specification**

- Model type: Random-effects Bayesian model
- Implementation: Conducted using the gemtc and rjags packages in R (version 4.5.1)
- Prior distribution:

Treatment effects: Normal(0, 15 × MLE variance)

Heterogeneity parameter (τ): Uniform(0, 5)

- Iterations: Four parallel Markov chains; 20,000 iterations per chain with a 5,000 burn-in
- Convergence diagnostics:

Assessed using the Brooks–Gelman–Rubin statistic (PSRF < 1.05)

Visual inspection of trace and density plots confirmed satisfactory convergence.

**3. Consistency and Transitivity Assessment**

**3.1 Transitivity Assumption**

The transitivity assumption was evaluated a priori by comparing the clinical and methodological characteristics of all included studies to ensure comparability.

No major differences were observed in:

- Baseline patient demographics (age, gender, disease severity)
- Intervention implementation (dosage, duration, co-interventions)
- Outcome definitions (mechanical ventilation duration, ICU IOS, PaO₂/FiO₂ ratio)

Therefore, transitivity was considered acceptable.

**3.2 Consistency Assumption**

The consistency assumption—agreement between direct and indirect evidence—was tested using both global and local methods:

- Global consistency test: Design-by-treatment interaction model [18]
- Local consistency test: Node-splitting method [19]
- Interpretation criterion: *P* > 0.05 indicates no statistically significant inconsistency.

Results summary:

- Global test (Design-by-treatment model): *P* > 0.05, indicating overall consistency.
- Node-splitting results: All *P* > 0.05 across pairwise comparisons (see Supplementary Figure 5).

Hence, no meaningful inconsistency was detected in the network.

**4. Software and Reproducibility**

- All analyses were conducted in R (version 4.5.1) using gemtc (v1.1-0) and rjags (v4-17), and validated in STATA (version 18.0; StataCorp, College Station, TX, USA).
- All scripts and model specification files are provided as Supplementary Files 2.

**Supplementary File 2. R Analysis Script (Representative Example)**

# Supplementary R script for Bayesian NMA

library(gemtc)

library(rjags)

# 1. Load dataset

data <- read.csv("ARDS_NMA_data.csv")

# 2. Construct network

network <- mtc.network(data.ab = data, description = "CHM interventions for ARDS")

# 3. Define model

model <- mtc.model(network,

type = "consistency",

linearModel = "random",

hy.prior = mtc.hy.prior("std.dev", "dunif", 0, 5),

n.chain = 4)

# 4. Run model

result <- mtc.run(model, n.adapt = 5000, n.iter = 20000)

# 5. Assess convergence

gelman.diag(result)

plot(result)

# 6. Check local inconsistency (node-splitting)

nodesplit <- mtc.nodesplit(network)

summary(nodesplit)

# 7. Calculate SUCRA rankings

ranks <- rank.probability(result)

sucra <- sucra(ranks)

plot(sucra)

# 8. Export results

write.csv(summary(result), "NMA_results.csv")

write.csv(summary(nodesplit), "Nodesplit_results.csv")
