# Supplementary material for: Efficacy comparison of four different Chinese herbal mediciness in intervening acute respiratory distress syndrome: a bayesian network meta-analysis
Source: Front Pharmacol. 2025 Nov 21;16:1671930. doi: 10.3389/fphar.2025.1671930 (PMC12678923; doi:10.3389/fphar.2025.1671930)
Supplement: Supplementary file 2 [file Table1.docx]

Supplementary Table 1. Standardized composition and taxonomic validation of included CHM formulas

(Standardized according to the Chinese Pharmacopoeia (2020 edition); botanical names verified in Plants of the World Online and World Flora Online.)

| Formula (Abbrev.) | English / Chinese Name | Composition — Latin binomial + Authority (Family) — Pharmacopoeial drug name |
| --- | --- | --- |
| Xuanbai Chengqi Decoction (XBCQD) | Xuanbai Chengqi Tang / 宣白承气汤 | Prunus armeniaca L. (Rosaceae) — Armeniacae Semen Amarum (Ku Xingren); Rheum palmatum L. (Polygonaceae) — Rhei Radix et Rhizoma (Dahuang); Trichosanthes kirilowii Maxim. (Cucurbitaceae) — Trichosanthis Pericarpium (Gualoupi); Gypsum Fibrosum (mineral origin, Shigao). Some variations additionally include Natrii Sulfas (mineral origin, Mangxiao). |
| Dachengqi Decoction (DCQD) | Da Chengqi Tang / 大承气汤 | Rheum palmatum L. (Polygonaceae) — Rhei Radix et Rhizoma (Dahuang); Natrii Sulfas (mineral origin, Mangxiao); Magnolia officinalis Rehder & E.H.Wilson (Magnoliaceae) — Magnoliae Officinalis Cortex (Houpo); Citrus aurantium L. (Rutaceae) — Aurantii Fructus Immaturus (Zhishi). |
| Liangge Powder (LGP) | Liangge San / 凉膈散 | Forsythia suspensa (Thunb.) Vahl (Oleaceae) — Forsythiae Fructus (Lianqiao); Rheum palmatum L. (Polygonaceae) — Rhei Radix et Rhizoma (Dahuang); Glycyrrhiza uralensis Fisch. ex DC. (Fabaceae) — Glycyrrhizae Radix et Rhizoma (Gancao); Gardenia jasminoides J.Ellis (Rubiaceae) — Gardeniae Fructus (Zhizi); Scutellaria baicalensis Georgi (Lamiaceae) — Scutellariae Radix (Huangqin); Mentha haplocalyx Briq. (Lamiaceae) — Menthae Herba (Bohe); Lophatherum gracile Brongn. (Poaceae) — Lophatheri Herba (Danzhuye); Natrii Sulfas (mineral origin, Mangxiao). |
| Fusu Agent (FSA) | Fusu Agent / 复苏合剂 (Resuscitation Formula) | Aconitum carmichaelii Debeaux (Ranunculaceae) — Aconiti Lateralis Radix Praeparata (Processed Aconite Root, Zhifuzi); Carapax Testudinis (animal origin, Guijia); Amomum villosum Lour. [≡ Wurfbainia villosa (Lour.) Skornick. & A.D.Poulsen] (Zingiberaceae) — Amomi Fructus (Sharen); Zingiber officinale Roscoe (Zingiberaceae) — Zingiberis Rhizoma (Ganjiang/Shengjiang, depending on preparation); Glycyrrhiza uralensis Fisch. ex DC. (Fabaceae) — Glycyrrhizae Radix Praeparata (Zhigancao); Ephedra sinica Stapf (Ephedraceae) — Ephedrae Herba (Mahuang). |

Notes:

Botanical identifications and authorities were verified through Plants of the World Online (POWO, Kew Science) and World Flora Online (WFO).

Non-botanical components (minerals or animal products) are explicitly indicated.

Where the original study did not specify the exact plant species, identities were standardized according to the Chinese Pharmacopoeia (2020 edition).
