# Supplementary figures and images for: Efficacy comparison of four different Chinese herbal mediciness in intervening acute respiratory distress syndrome: a bayesian network meta-analysis
Source: Front Pharmacol. 2025 Nov 21;16:1671930. doi: 10.3389/fphar.2025.1671930 (PMC12678923; doi:10.3389/fphar.2025.1671930)

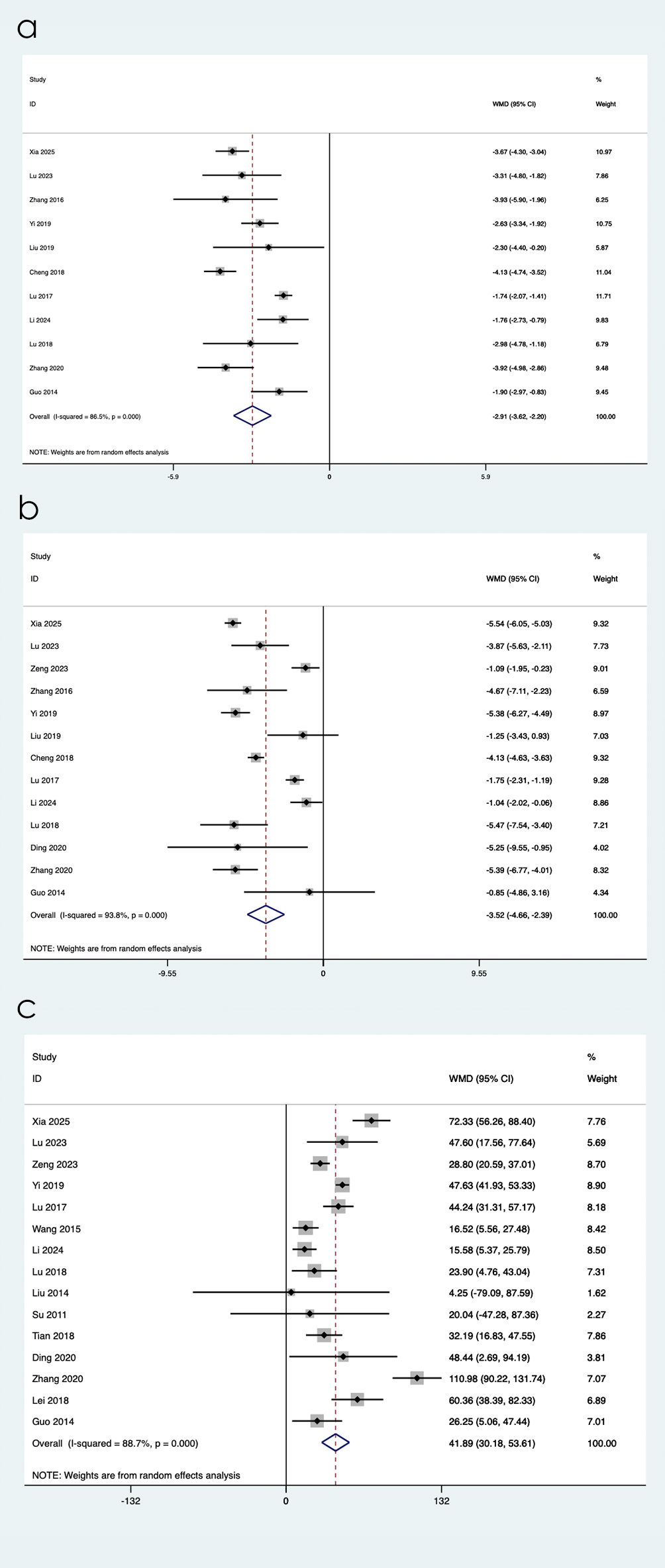

Supplement: Supplementary file 3 [file Image3.tif]

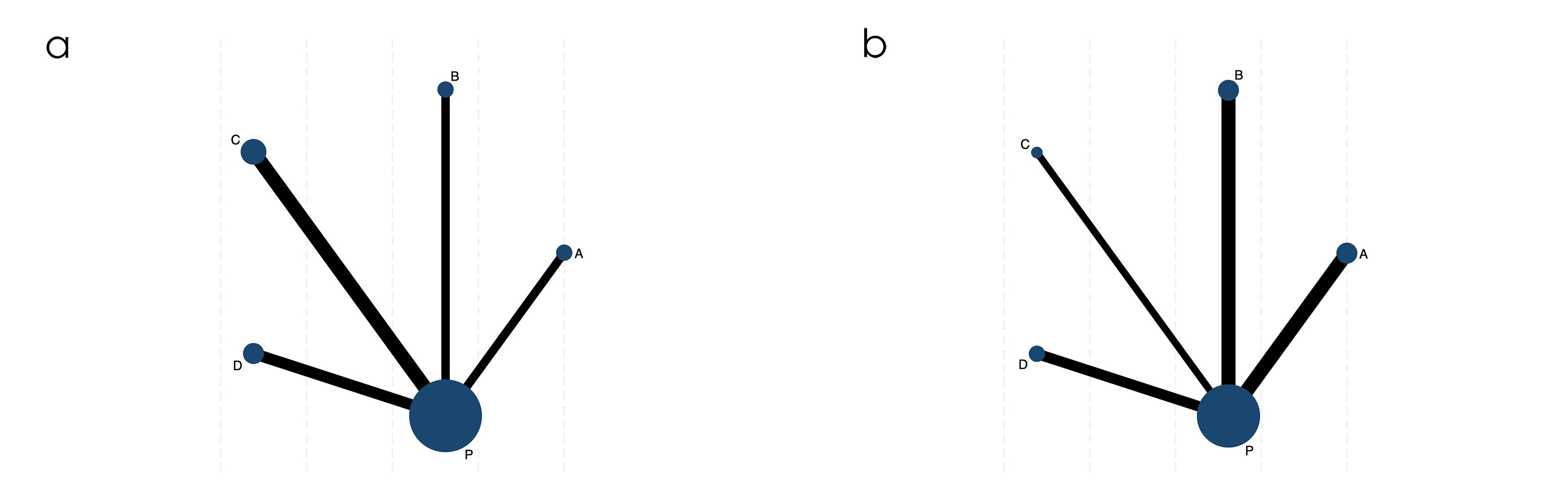

Supplement: Supplementary file 4 [file Image4.tif]

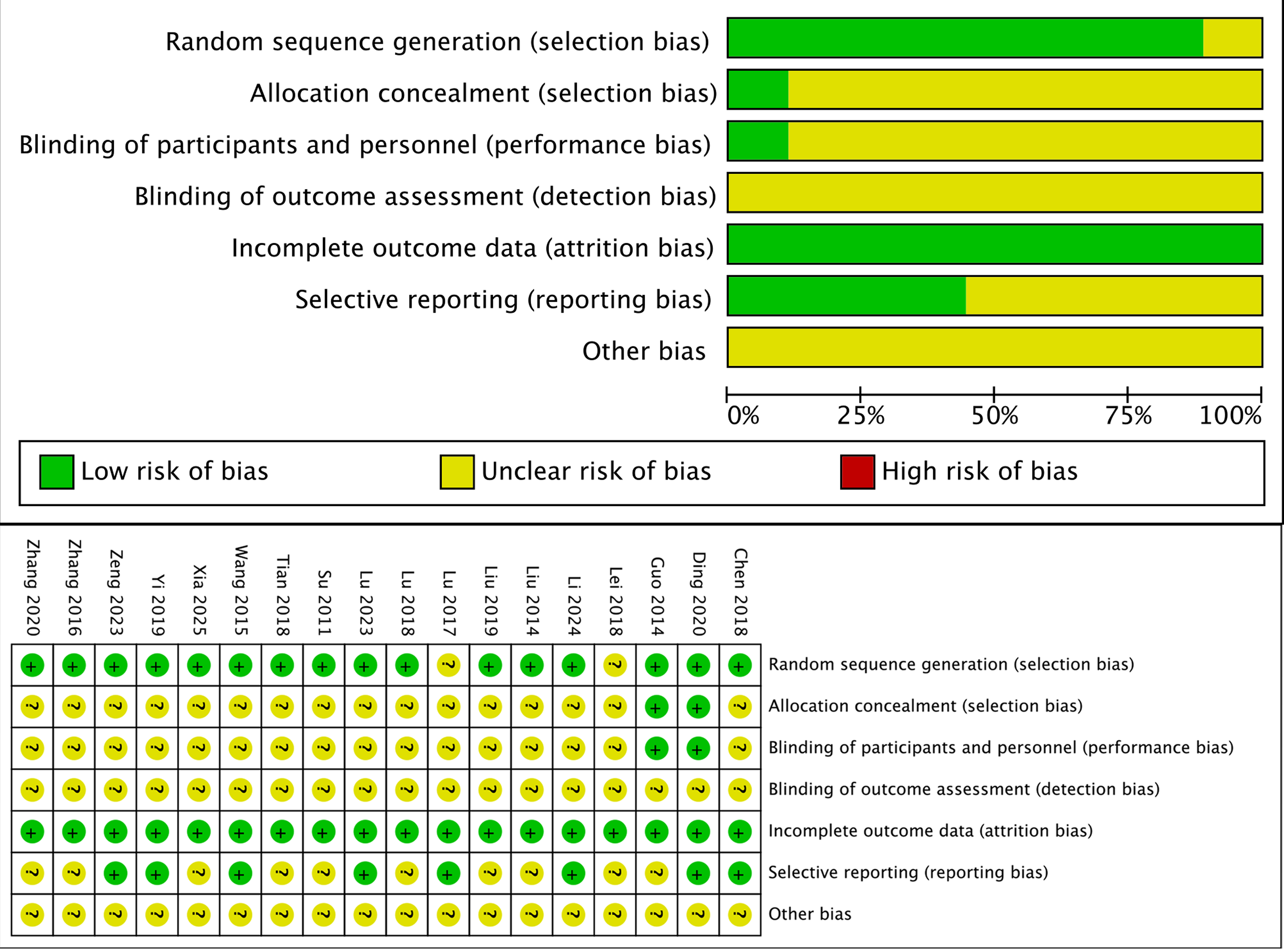

Supplement: Supplementary file 5 [file Image2.tif]

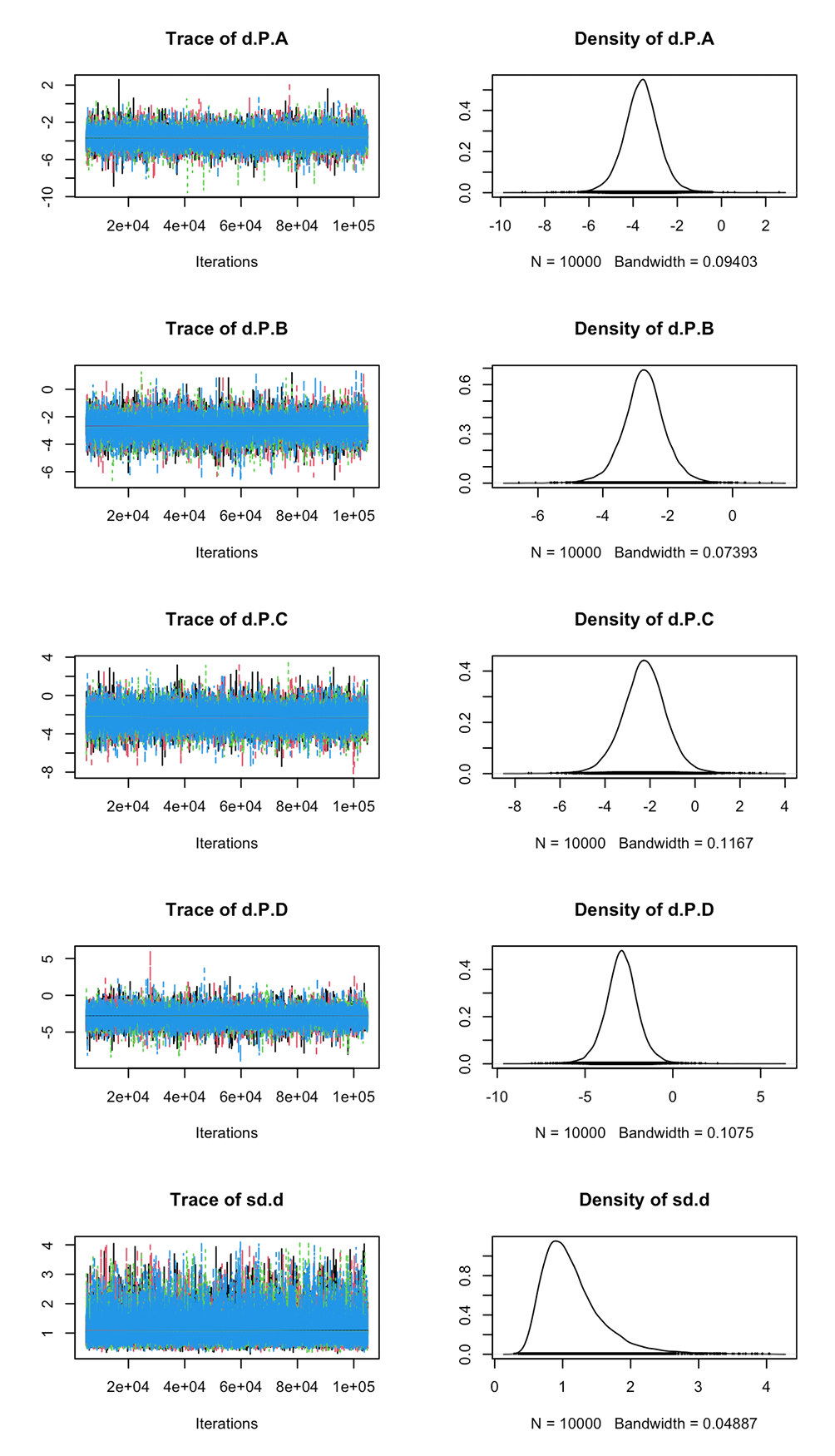

Supplement: Supplementary file 6 [file Image1.tif]

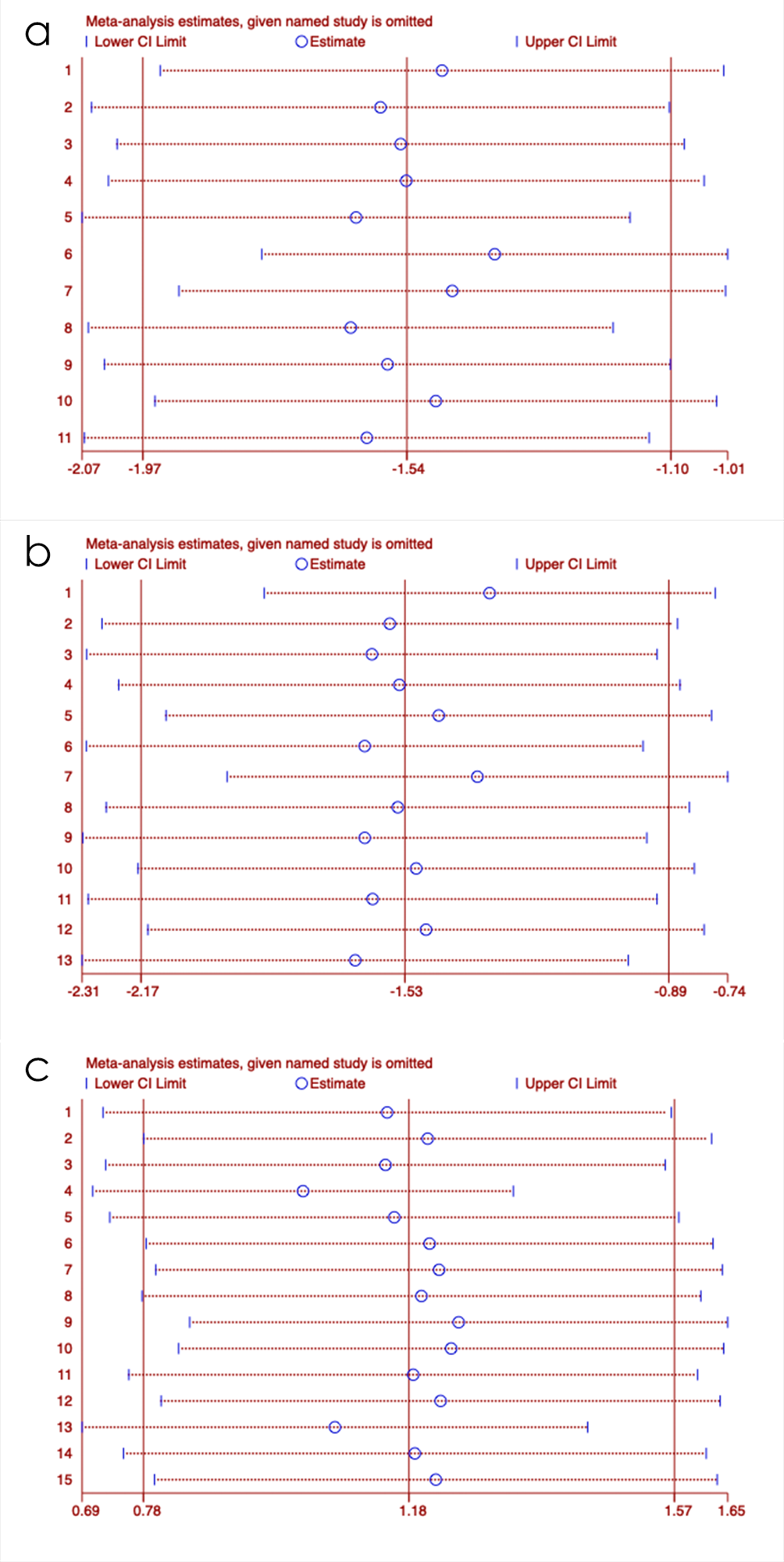

Supplement: Supplementary file 7 [file Image7.tif]

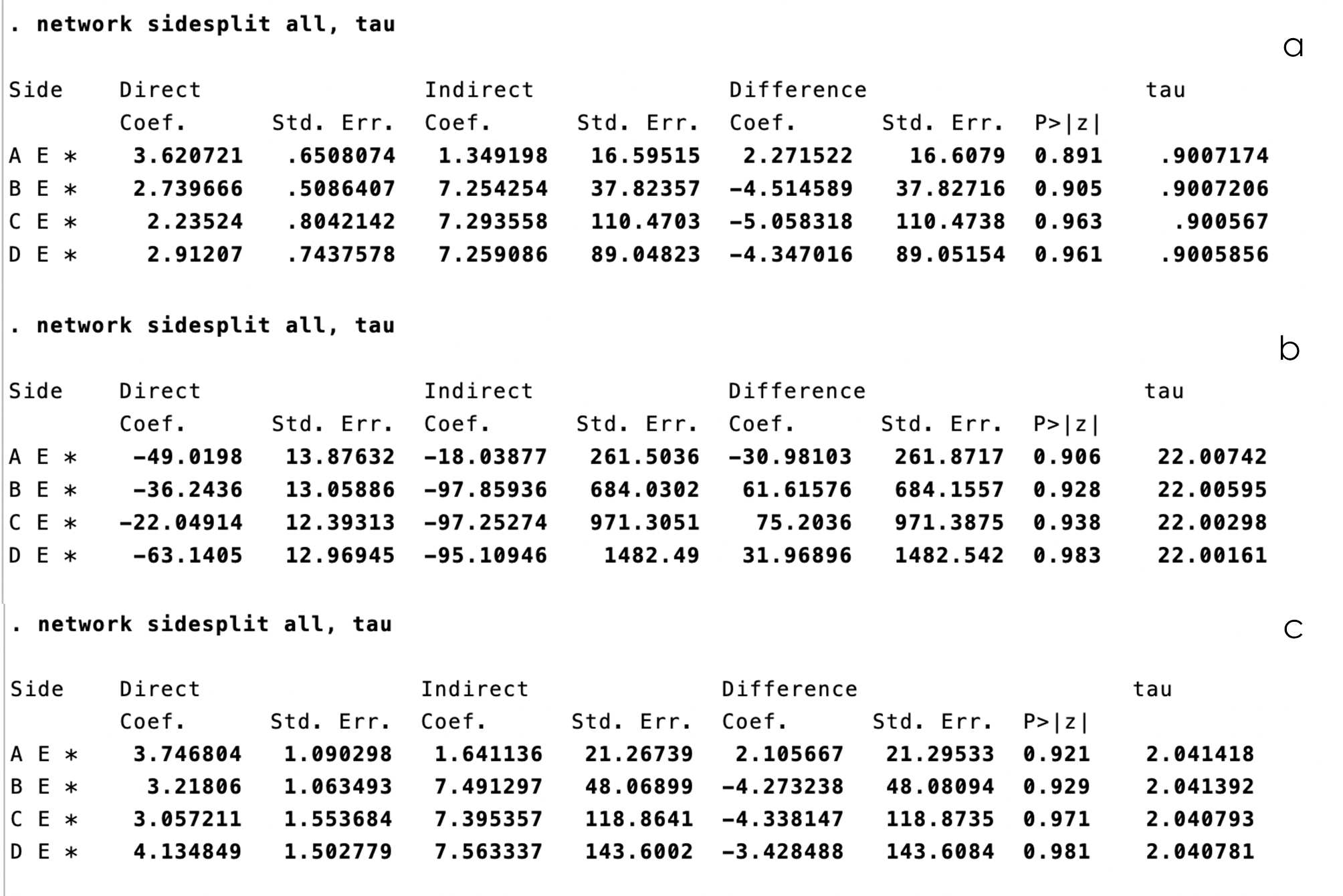

Supplement: Supplementary file 11 [file Image5.tif]

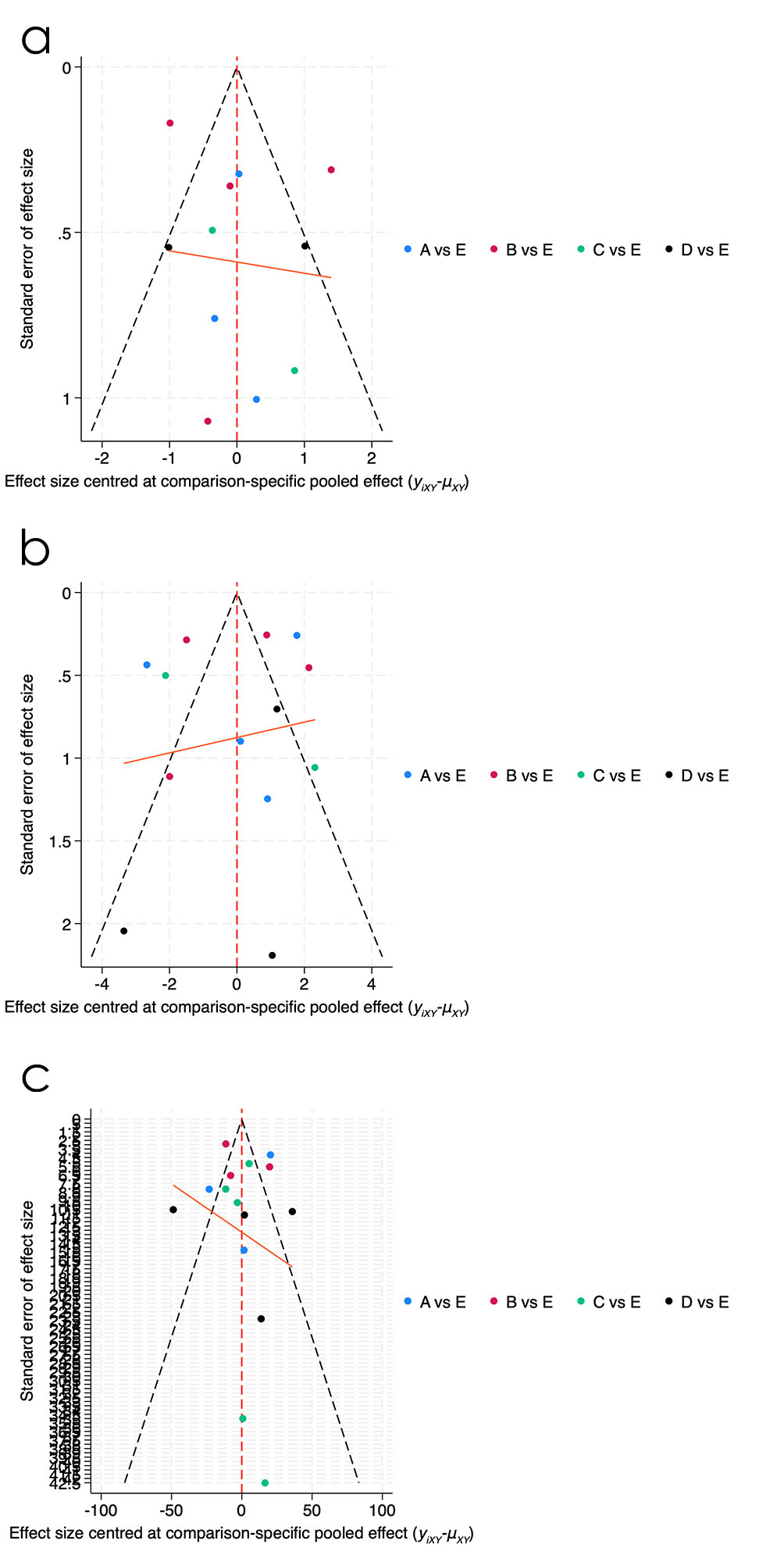

Supplement: Supplementary file 12 [file Image6.jpeg]
