# Supplementary material for: Efficacy comparison of four different Chinese herbal mediciness in intervening acute respiratory distress syndrome: a bayesian network meta-analysis
Source: Front Pharmacol. 2025 Nov 21;16:1671930. doi: 10.3389/fphar.2025.1671930 (PMC12678923; doi:10.3389/fphar.2025.1671930)
Supplement: Supplementary file 8 [file Table2.docx]

Supplementary Material Table 2. The search strategy for PubMed.

| Number | Search terms |
| --- | --- |
| #1 | "acute respiratory distress syndrome" [Mesh] OR "ARDS" [Title/Abstract] |
| #2 | "Xuanbai Chengqi Decoction" [Title/Abstract] OR "Xuanbai Chengqi Tang" [Title/Abstract] OR "XBCQD"[Title/Abstract] OR "Dachengqi Decoction" [Title/Abstract] OR "Dachengqi Tang" [Title/Abstract] OR "DCQD" [Title/Abstract]) OR "Liangge Powder" [Title/Abstract] OR "Liangge San" [Title/Abstract] OR "LGP" [Title/Abstract]) OR "Fusu Agent" [Title/Abstract] OR "Fusuji" [Title/Abstract] OR "FSA"[Title/Abstract] |
| #3 | "Randomized Controlled Trial" [Publication Type] OR "RCT randomized controlled" [Publication Type] OR "random allocation" [Title/Abstract] OR "allocation, random" [Title/Abstract] OR "randomized, controlled" [Title/Abstract] OR "clinical trial"[Title/Abstract] |
| #4 | #1 AND #2 AND #3 |
