# Supplementary material for: Efficacy comparison of four different Chinese herbal mediciness in intervening acute respiratory distress syndrome: a bayesian network meta-analysis
Source: Front Pharmacol. 2025 Nov 21;16:1671930. doi: 10.3389/fphar.2025.1671930 (PMC12678923; doi:10.3389/fphar.2025.1671930)
Supplement: Supplementary file 9 [file Table3.docx]

Supplementary Material Table 3a. Meta-regression for MVD

_ES | Coef. Std. Err. t P>|t| [95% Conf. Interval]

intervention time | .3576432 .3936517 0.91 0.390 -.5501192 1.265406

administration | -.0883069 .4005403 -0.22 0.831 -1.011954 .8353406

_cons | -3.394341 1.299405 -2.61 0.031 -6.390774 -.3979074

Supplementary Material Table 3b. Meta-regression for ICU LOS

_ES | Coef. Std. Err. t P>|t| [95% Conf. Interval]

intervention time | .2019706 .6929114 -0.29 0.777 -1.745873 1.341932

administration | -.9137486 .6777369 -1.35 0.207 -2.423841 .5963433

_cons | -1.443225 2.052092 -0.70 0.498 -6.015571 3.129122

Supplementary Material Table 3c. Meta-regression for P/F ration

_ES | Coef. Std. Err. t P>|t| [95% Conf. Interval]

intervention time | 9.71707 7.525114 1.29 0.221 -6.678745 26.11288

administration | -12.87964 8.180454 -1.57 0.141 -30.70332 4.944035

_cons | 49.14244 23.20862 2.12 0.056 -1.42479 99.70967
