# Supplementary material for: Efficacy comparison of four different Chinese herbal mediciness in intervening acute respiratory distress syndrome: a bayesian network meta-analysis
Source: Front Pharmacol. 2025 Nov 21;16:1671930. doi: 10.3389/fphar.2025.1671930 (PMC12678923; doi:10.3389/fphar.2025.1671930)
Supplement: Supplementary file 10 [file Table4.docx]

Supplementary Material Table 4a. Egger's test for MVD

Std_Eff | Coef. Std. Err. t P>|t| [95% Conf. Interval]

slope | -.1.949486 .5754481 -3.39 0.008 -3.25124 -.6477318

bias | -1.942408 1.532838 -1.27 0.237 -5.409928 1.525112

Supplementary Material Table 4b. Egger's test for ICU LOS

Std_Eff | Coef. Std. Err. t P>|t| [95% Conf. Interval]

slope | -3.906448 .9685162 -4.03 0.002 -6.038138 -1.774758

bias | .6459386 2.122404 0.30 0.767 -4.02544 5.317318

Supplementary Material Table 4c. Egger's test for P/F ration

Std_Eff | Coef. Std. Err. t P>|t| [95% Conf. Interval]

slope | 36.3158 9.615791 3.78 0.002 15.54215 57.08945

bias | .4439663 1.45104 0.31 0.764 -2.690815 3.578747
